# Short-Term Restriction of Physical and Social Activities Effects on Brain Structure and Connectivity

**DOI:** 10.3390/brainsci15010007

**Published:** 2024-12-25

**Authors:** Yajuan Zhang, Lianghu Guo, Zhuoyang Gu, Qing Yang, Siyan Han, Han Zhang

**Affiliations:** 1 School of Biomedical Engineering and State Key Laboratory of Advanced Medical Materials and Devices, ShanghaiTech University, Shanghai 201210, China; zhangyj5@shanghaitech.edu.cn (Y.Z.); guolh@alumni.shanghaitech.edu.cn (L.G.); guzhy@shanghaitech.edu.cn (Z.G.); hansy2022@shanghaitech.edu.cn (S.H.); 2 Shanghai Clinical Research and Trial Center, Shanghai 201210, China

**Keywords:** lockdown environments, longitudinal study, resting-state fMRI, structural MRI, functional connectivity

## Abstract

Background: Prolonged confinement in enclosed environments has raised concerns about its effects on both physical and mental health. Although increased rates of depression or anxiety during COVID-19 lockdowns have been reported, the effects of short-term restrictions on social activities and physical on brain function and structure remain poorly known. Methods: This study explored longitudinal changes in brain gray matter volume (GMV) and functional connectivity (FC) immediately after and four months following a short-term lockdown in comparison to pre-lockdown conditions. MRI data were collected from 20 participants before the lockdown, from 29 participants (14 original, 15 new) two months post-lockdown, and from 27 out of the 29 participants four months post-lifting of the lockdown. Results: Results showed significant GMV reductions in the right gyrus rectus and cuneus post-lockdown, with further reductions observed four months after lifting the restrictions, affecting additional brain regions. Longitudinal FC trajectories revealed decreased connectivity between the default mode network (DMN) and sensorimotor/attention networks post-lockdown, and recovery after four months post-lifting of the lockdown. Conclusions: The observed plasticity in brain FC indicates substantial recovery potential with the potential long-term effect of structural changes. Our findings offer insights into the effects of isolation on the human brain, potentially informing rehabilitation mechanisms and interventions for individuals in similar conditions.

## 1. Introduction

People isolated from social contact were prone to experience mental and physical health issues, such as stress [1], anxiety [2,3,4], and depression [5,6]. Throughout the coronavirus disease 2019 (COVID-19) pandemic, despite the use of population-wide lockdowns as an effective quarantine strategy to reduce morbidity and mortality [7,8,9], these lockdowns contributed to the adverse psychological impacts observed in isolated individuals [10]. Compared to the general population, university students are particularly vulnerable to mental health challenges due to the stress associated with academic demands, societal expectations, financial constraints, and the experience of isolation [11]. The lockdown during the COVID-19 pandemic presents a rare opportunity to examine the impact of these factors on brain health in young adults.

Depression, anxiety, distress, and fear are prominent emotional responses commonly associated with the implementation of strict lockdown measures. These emotional reactions may further cause emotional dysregulation and negative social behaviors [12]. Recent evidence from a systematic review shows that the fear of viral infections and stringent social distancing measures lead to increased negative emotions and loneliness [13]. Furthermore, it is well-established that physical activity plays a crucial role in promoting brain health, facilitating neuroplasticity, and inducing acute and sustained psychophysiological effects, including mood improvements [14,15], stress reduction, and decreased anxiety [16]. Physical inactivity, such as sedentary behavior, and the lack of social engagement during the pandemic may negatively affect both physical and mental well-being. Emerging evidence also suggests a negative impact of lockdown measures on cognitive and mental functioning [17,18,19].

Over the years, neuroimaging research has demonstrated the significant potential for external experiences to induce changes in the adult brain [20]. Investigations have shown that learning can induce measurable alterations in brain structure as observed in structural magnetic resonance imaging (MRI) [21,22,23]. A recent study involving healthy adults revealed significant increases in gray matter volume (GMV) in the ventral anterior temporal cortices, amygdala, and putamen, which were linked to pandemic-related stress and anxiety following the COVID-19 outbreak and lockdown [24]. Psychological stressors have been demonstrated to exert broad effects on both the brain and behavior [25,26], with responses to psychosocial stressors initiating in higher brain regions such as the prefrontal cortex (PFC) and interconnected limbic nuclei [27]. In animal studies, similarity finding also indicated that stressful experiences can influence both the structure and function of the PFC. Nevertheless, this impact is not enduring, as young animals exhibit notable neural resilience once the stressors cease [25]. Additionally, functional MRI (fMRI), which measures brain activity through blood oxygenation level-dependent signals, can aid in the identification of activity and connectivity changes associated with environmental factors [28,29]. Studies have shown that healthy participants exhibited decreased FC between the sensorimotor network and the default mode network (DMN) after reducing sensory inputs from the environment, and such a reduction was associated with a significant improvement in depression and anxiety levels [28,30,31]. Such a study raises concerns about the potential structural and functional brain changes resulting from prolonged isolation, reduced physical and social activities, and increased depression and anxiety levels.

In this study, we aim to evaluate the effect of physical lockdown and social isolation on brain function and structure in college students and whether lifting of the lockdown measures could result in full/partial recovery of brain alterations or have a long-lasting effect. Longitudinal changes in the brain GMV and the whole-brain FC were investigated before a full two-month lockdown, immediately after the lockdown, and four months after the lift of the lockdown measures. According to the existing evidence, we hypothesized that (i) abnormal GMV and brain FC would manifest after the lockdown, possibly due to physical inactivity, social isolation, and prolonged anxiety; and (ii) such alterations in the brain GMV and FC would be partially reversed after four months of deconfinement, possibly due to neuroplasticity. Our extended investigation into the influence of a constrained environment on the brain structure offers valuable perspectives on the repercussions of enduring isolation and decreased physical/social engagements on the human brain.

## 2. Materials and Methods

### 2.1. Participants

A total of 20 healthy participants aged 18–27 were recruited from ShanghaiTech University. Prior to the COVID-19 lockdown, participants underwent an initial MRI scan (Scan1). Three months later, participants experienced a full lockdown, which involved restricted physical and social activities under strict quarantine protocols, including confinement in dormitory conditions for approximately two months. Following the two-month lockdown, 29 participants (14 original and 15 new) were recruited for a second MRI scan (Scan2, two months after the lockdown) to examine the effects of the lockdown. During the lockdown period, we used online advertising and communication channels to reach individuals who had expressed interest in the study, and these 15 new participants were recruited. Following the lifting of the lockdown measures, 27 out of the 29 participants underwent a follow-up MRI scan (Scan3) four months later to examine the potential long-lasting effects. Participants were included if they did not have a history of significant head injury, neurological diseases, mental disorders, or significant medical conditions such as hepatic or renal diseases, or severe cardiovascular, and if they did not have standard MRI contraindications such as current pregnancy, metal implants, breastfeeding, or claustrophobia.

### 2.2. Mental Status Measurement

Participants were asked to complete the mental status assessments before the MRI scans at both Scan 2 and Scan 3. This timing allowed for the assessment of mental health status prior to the brain imaging. At the Scan2 and Scan3, participants were assessed using the 14-item Perceived Stress Scale (PSS-14) [32] (score range: 0–56), General Anxiety Disorder scale-7 (GAD-7) [33] (score range: 7–21), Patient Health Questionnaire-9 (PHQ-9) [34] (score range: 5–36), and the Positive and Negative Affect Scale (PANAS-P, score range: 0–50; PANAS-N, score range: 0–50) [35]. Higher scores on the PSS-14, GAD-7, PHQ-9, and PANAS-P/N indicate a higher level of stress, anxiety, depression, or positive/negative affect symptoms. All these mental status scores were used to associate with the MRI findings.

### 2.3. MRI Data Acquisition

This experiment was carried out using a United Imaging 3.0T uMR890 scanner with a 64-channel head coil, manufactured by United Imaging Healthcare, Shanghai, China, at ShanghaiTech University. Subjects were positioned supine with inflatable pillows to minimize head movement during magnetic resonance scanning. Anatomical T1-weighted images for each participant were obtained by fast MRI acquisition technique using united Compressed Sensing (uCS) with an acceleration factor of 3.32. The T1-weighted structural MRI (sMRI) parameters were as follows: repetition time (TR) = 7.4 ms; echo time (TE) = 3.4 ms; field of view (FOV) = 256 × 240 mm^2^; flip angle (FA) = 8°; resolution = 0.8 × 0.8 mm^2^; slice number = 208; and slice thickness = 0.8 mm. During the resting-state fMRI (rs-fMRI) scan, all participants were asked to remain awake with their eyes closed, not think systematically, and move as little as possible. The rs-fMRI parameters were as follows: TR = 800 ms; TE = 35 ms; acceleration of multiband factor = 4; FOV = 209 × 209 mm^2^; slice thickness = 1.8 mm; resolution = 1.8 × 1.8 mm^2^; number of axial slices = 72; matrix = 116 × 116; flip angle = 52°; and total volumes measurement = 450. Additionally, we obtained a single-band image with the same parameters for use as a reference image. Two-phase encoding directions (P→A and A→P) were collected using the EPI sequence for distortion correction.

### 2.4. MRI Data Processing

All T1-weighted imaging data were preprocessed and analyzed using the Computational Anatomy Toolbox (CAT12, http://dbm.neuro.uni-jena.de/cat/, accessed on 1 April 2023) and Statistical Parametric Mapping toolbox (SPM12, http://www.fil.ion.ucl.ac.uk/spm, accessed on 1 April 2023). The procedure includes the following steps: (1) denoising with a spatial adaptive non-local means (SANLM) filter [36]; (2) bias correction and affine registration [37]; (3) skull-stripping and parcellation of the brain; (4) local intensity transformation of all tissue classes; (5) refinement of the adaptive maximum a posteriori (AMAP) segmentation [38]; (6) normalization of the tissues to the MNI space using Geodesic Shooting registrations; and (7) application of the inverse warp generated to bring the MNI-space atlas into native spaces for each subject. Subsequently, the automated anatomical labeling (AAL2 [39]) atlas was used to calculate the GMV in each of the 120 cortical and subcortical regions of interest (ROIs) for each subject.

The preprocessing of the rs-fMRI data were performed using the FMRIB software library (FSL v6.0, https://fsl.fmrib.ox.ac.uk/fsl/fslwiki, accessed on 1 April 2023) and Advanced Normalization Tools (ANTS) [40]. The preprocessing procedure comprised the following steps: (1) motion correction with rigid-body alignment and spatial distortion correction; (2) registration of rs-fMRI scans to their corresponding T1 image via boundary-based registration [40]. Specifically, the rs-fMRI scans were aligned to the T1 image of the same subject with the tissue segmentation maps used as guidance. The accuracy of the co-registration was finally confirmed through visual inspection; (3) resampling of fMRI data in the native spaces; (4) decomposition of each fMRI signal into 150 independent components by MELODIC in FSL and removal of noisy components [41]. Additional regressors included the Friston-24 head motion parameters; and (5) a temporal band-pass filter. A quality control approach based on carpet maps was implemented to ensure the quality of rs-fMRI preprocessing.

The preprocessed rs-fMRI data were further parcellated using the AAL2 atlas [39]. The averaged rs-fMRI time series for all voxels within each region was extracted. A 120 × 120 FC matrix was then derived by calculating Pearson’s correlation coefficient between the time series courses of each pair of brain regions. Fisher’s *r*-to-*z* transformation was applied to improve the normality of the FC. To define functional networks, we assigned the AAL2 regions to Yeo’s seven functional networks based on the highest overlapping ratio (percentage of voxels of each region within each network) [42]. The subcortical areas were included as a separate network. The results of the FC analysis were visualized using Circos (http://circos.ca/software/download/, accessed on 1 May 2023) and BrainNet Viewer (https://www.nitrc.org/projects/bnv/, accessed on 1 May 2023).

### 2.5. Statistical Analysis

The differences in mental status as measured by PSS-14, GAD-7, PHQ-9, and PANAS-P/N between Scan2 and Scan3 were calculated as indices of affected mental health due to lockdown and compared using a paired *t*-test (two-tailed, with a significance level of *p* < 0.05).

Brain structural and functional changes were examined by comparing the three groups using paired *t*-tests. That is, changes in GMV and FC measures before, right after, and four months after lockdown were separately investigated. Specifically, (i) for the GMV analysis, pairwise *t*-tests were conducted for Scan1 vs. Scan2, Scan1 vs. Scan3, and Scan2 vs. Scan3 (*p* < 0.05, false discovery rate (FDR) correction); (ii) for the whole brain FC, a network-based statistical approach (NBS) was used for Scan1 vs. Scan2, Scan1 vs. Scan3, and Scan2 vs. Scan3 (cluster-defining threshold, *p* < 0.05, random permutation, 5000, the significance level of the connected components, *p* < 0.05); (iii) for the whole brain FC, longitudinal analysis with missing data were also conducted using nonlinear mixed-effects models (NLME) in Python to delineate the longitudinal trajectories [43]. In the NLME model, FC was set as a dependent variable, and MRI scan time points (Scan1, Scan2, Scan3), mean framewise displacement (FD), and sex were independent variables. We included random intercepts and subject effects as random effects. Significant longitudinal changes were identified with a threshold of *p* < 0.01 after applying multiple comparison correction using the FDR.

After identifying differences in GMV and FCs between groups, we conducted a series of correlation analyses to explore potential associations of the MRI findings with mental health measures. Specifically, utilizing the observed between-group differences in GMV and FCs, a series of partial correlation analyses were conducted to investigate the associations between alterations in GMV and FC and improvements in PSS-14, GAD-7, PHQ-9, and PANAS-P/N scores. Age, gender, and years of education were included as covariate variables to control for their potential effects.

## 3. Results

### 3.1. Demographic and Clinical Characteristics

**Figure 1:**
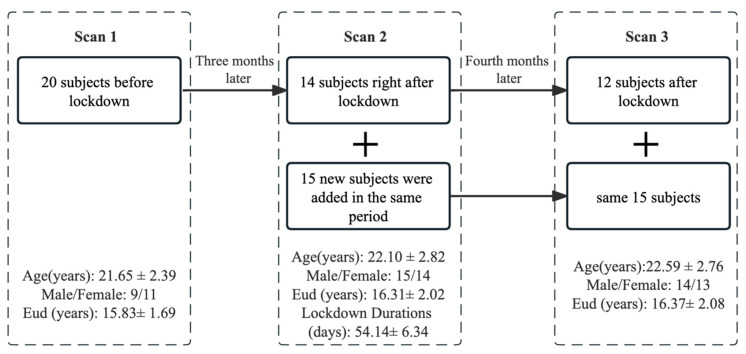
Flowchart for participants’ scans. (i) A total of 20 healthy adults aged 18–27 were recruited, and MRI scans; (ii) three months later, participants were placed in a full lockdown environment. Right after lockdown, a total of 14 original participants and 15 new participants were enrolled for MRI scans and clinical assessments; and (iii) four months following the lifting of lockdown measures, 27 of the 29 participants underwent a third follow-up MRI scan and clinical assessment.

Figure 1 shows the demographics for the participants in three MRI scan time points. The COVID-19 lockdown durations were 54.14 ± 6.34 days. There were no significant differences in PANAS-P (*p* = 0.058) and PANAS-N (*p* = 0.7) between the participants Scan2 and Scan3. The PSS14 scores (Scan2: 27.5 ± 9.5; Scan3: 22.0 ± 6.8, *p* = 0.0057), GAD7 scores (Scan2: 6.3 ± 4.8; Scan3: 3.7 ± 2.4, *p* = 0.0239), and PHQ9 scores (Scan2: 8.0 ± 4.7; Scan3: 4.2 ± 3.0, *p* = 0.0007) were significantly decreased in Scan3 compared to Scan2, as revealed by the paired *t*-test (Table 1).

### 3.2. Regional-Based Comparison of GMV

**Figure 2:**
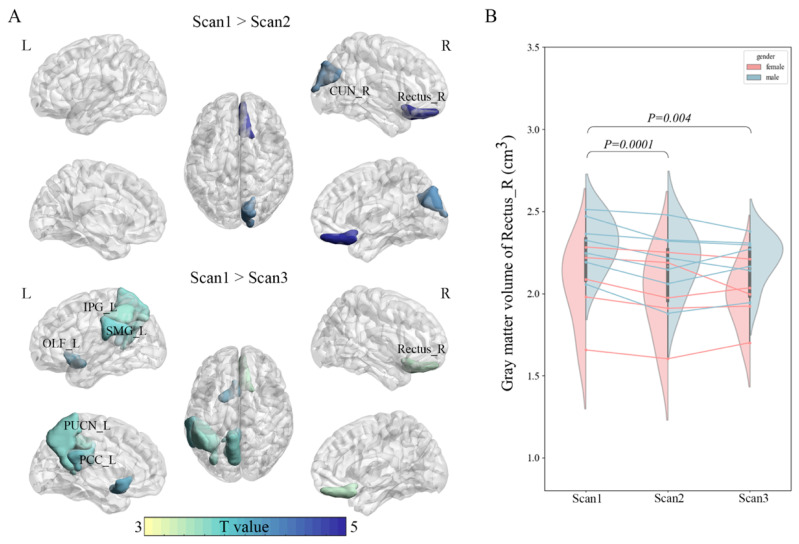
Group differences of gray matter volume. (**A**) Regions with significant differences in GMV between Scan1 and Scan2 (**upper**) and between Scan1 and Scan3 (paired *t*-test, *p* < 0.05, FDR correction). (**B**) Longitudinal alteration in the GMV in the right gyrus rectus between groups. There was a significant decrease in GMV in Scan2 and Scan3 compared to Scan1 (Scan1 > Scan2: *p* = 0.0001; Scan1 > Scan3: *p* = 0.004; paired *t*-test).

Figure 2 and Table 2 showed the results of pairwise group comparison for the GMV. Compared with Scan1, participants exhibited significantly decreased GMV in the right gyrus rectus and right cuneus (paired *t*-test, *p* < 0.05, FDR correction) in Scan2. Similarly, participants four months after the lockdown period (Scan3) showed a significant reduction in GMV in the left rolandic operculum, supramarginal gyrus, inferior parietal, posterior cingulate gyrus, precuneus, and right gyrus rectus compared to the pre-lockdown period (Figure 2A, paired *t*-test, *p* < 0.05, FDR correction). Longitudinal alteration in the GMV in the right gyrus rectus was found (Figure 2B, Scan1 > Scan2: *p* = 0.0001; Scan1 > Scan3: *p* = 0.004). There were no group differences between the Scan2 and Scan3. Additionally, no correlations between GMV and clinical variables (i.e., PSS-14, GAD-7, PHQ-9, and PANAS-P/N) were found.

### 3.3. Longitudinal Trajectories of Whole-Brain FC

**Figure 3:**
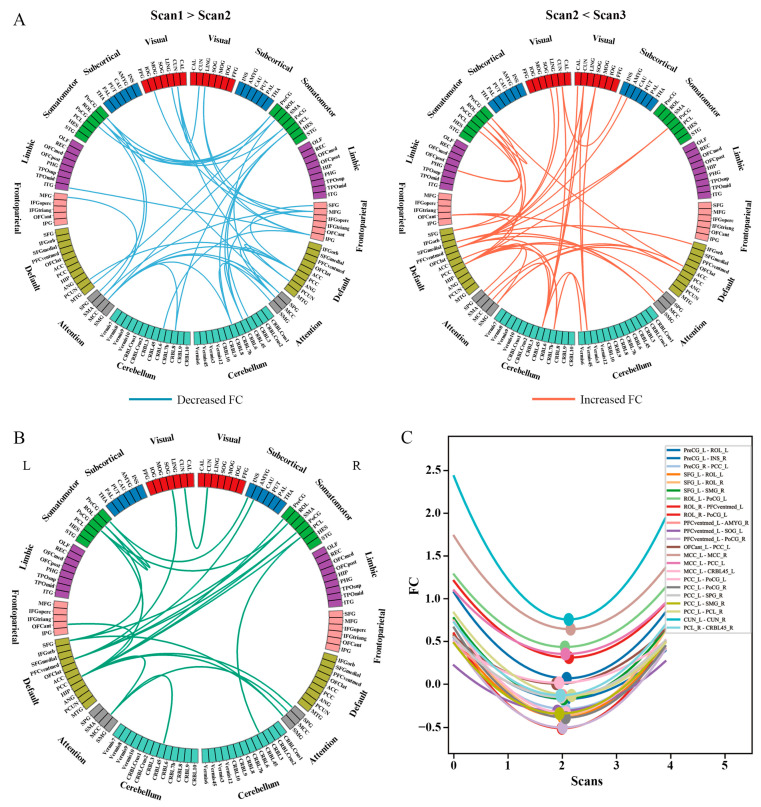
Group differences of functional connectivity. (**A**) Significantly decreased FC at Scan2 compared to Scan1 and significantly increased FC at Scan3 compared to Scan2. (**B**) The significant trajectories in the FC fit curves (*p* < 0.01, FDR-corrected). (**C**) These fit curves show that the FC decreased from pre-lockdown to right after lockdown and then increased from right after lockdown to post-lockdown. The color of the circle represents the 120 different regions from the Automatic Anatomical Labeling 2 (AAL2) atlas.

Figure 3A showed the results of the pairwise group comparisons for whole-brain FC. A subnetwork using the NBS approach (*p* < 0.05, 5000 permutation test, Figure 3A) was identified that demonstrated a decrease in FC at Scan2 compared to Scan1. The primary decrease in FC was observed between the attention network (ATN) (including bilateral superior occipital gyrus (SOG), median cingulate and paracingulate gyri (MCC), right supramarginal gyrus (SMG), and left supplementary motor area (SMA)) and the somatomotor network (SMN) (including bilateral precentral gyrus (PreCG), postcentral gyrus (PoCG), rolandic operculum (ROL), and right left paracentral lobule (PCL)), between visual network (VIS) (cuneus (CUN), lingual gyrus (LING), and fusiform gyrus (FFG)) and frontoparietal network (FPN) (including the right middle frontal gyrus (MFG), inferior parietal gyrus (IPG), and inferior frontal gyrus of opercular part (IFGoperc)), within ATN, and within SMN. Similarly, a subnetwork was identified (Figure 3B) that showed an increase in FC at Scan3 compared to Scan2. The primary increased FCs were observed between the default network (DMN) (including bilateral posterior cingulate gyrus (PCC), anterior cingulate and paracingulate gyri (ACC), and left superior frontal gyrus of medial orbital (PFCventmed)) and visual network (bilateral CUN, SOG, left LING, and right calcarine cortex (CAL)), SMN (including bilateral PoCG, left PreCG, ROL, PCL), and ATN. There were no group differences between Scan1 and Scan3.

Figure 3B showed the longitudinal trajectories of FC across all brain regions as estimated by the NLME model. The results demonstrate significant U-shaped trajectories in the FC fit curves between two primary subnetworks: the DMN and the SMN. Specifically, the U-shaped fit curves of the FC were mainly observed between the left PCC in the DMN and SMN (including bilateral PoCG, right PreCG, and PCL), ATN (including right SPG, SMG, and left MCC), between the left PFCventmed and SMN (including right PoCG and ROL), and SFG and bilateral ROL. The FC within the SMN also displayed U-shaped trajectories (Figure 3C, *p* < 0.01, FDR-corrected). These fit curves show that the FC decreased from Scan1 to Scan2, and then increased from Scan2 to Scan3 (Figure 3C).

### 3.4. The Association Between Longitudinal Altered FC and Mental Status Measurement Scores

**Figure 4:**
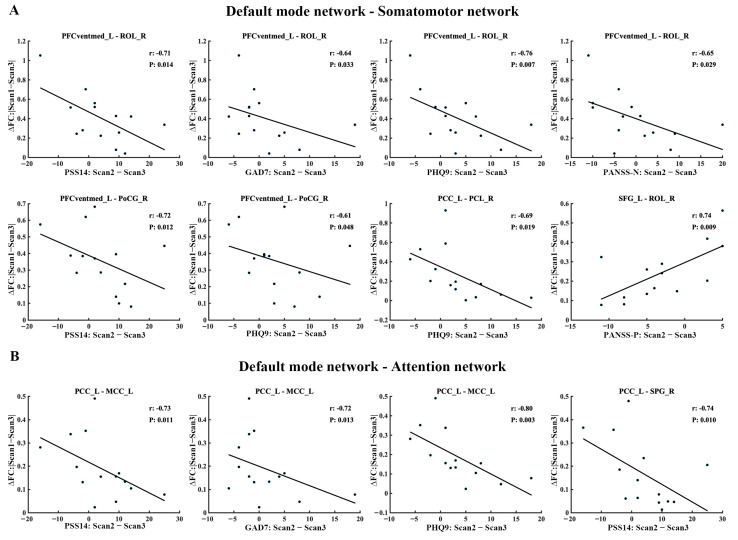
The correlation between longitudinally altered FC and mental status measurement scores. (**A**) The correlation between longitudinal changes in FC within the DMN and SMN and mental status measurement scores. (**B**) The correlation between longitudinal changes in FC within the DMN and ATN and mental status measurement scores. Partial correlation was used with controlling sex, age, and education of fMRI. The significance level was set at *p* < 0.05.

As shown in Figure 4, the absolute values of FC changes (Scan1–Scan3) between the DMN (including left PFCventmed, PCC, and SFG) and the SMN (including right ROL, PoCG, and PCL) showed a negative correlation with the improvement in mental status measurements such as PSS14, GAD7, PHQ9, and PANAS-N (positive correlation with improvement in PANAS-P), suggesting the greater proximity of FC in the third scan to baseline is associated with better symptom recovery (Figure 4A). Similarly, the absolute values of FC changes (Scan1–Scan3) between the DMN (including left PCC) and ATN (including left MCC and right SPG) showed a negative correlation with the improvement in mental status measurements in PSS14, GAD7, and PHQ9 (Figure 4B).

## 4. Discussion

This study explores how a brief period of restricted physical and social activities affects brain structure and function in young adults, revealing a notable macroscopic impact on the brain, potentially resulting from this experience. Images were collected in the longitudinal study design before, right after, and four months after the lockdown. The main findings are as follows: (1) Participants showed significantly decreased GMV in the right cuneus and gyrus rectus right after the lockdown period. Four months after the lockdown was lifted, additional brain regions showed notable volumetric decreases. (2) Right after the lockdown, the FC in a subnetwork including ATN, SMN, VIS, and FPN significantly decreased compared to pre-lockdown. After the lockdown lifting, FC in a subnetwork including VIS, SMN, and DMN significantly increased compared to right after the lockdown period. The further longitudinal analysis showed that right after the lockdown period, the FC between DMN and SMN/ATN, as well as FC within SMN, were significantly decreased, and this trend was reversed after four months of deconfinement. Furthermore, the recovery of FCs between DMN and SMN/ATN was found to be significantly correlated with the improvement of mental status measurements. These findings enhance our understanding of how prolonged isolation and reduced physical and social activities affect the human brain.

In addition to the direct neurological changes caused by COVID-19 infection [44,45,46,47,48,49,50], our findings indicate that the changes in brain morphology and function are also associated with the profound effects of the strictly enclosed environment. In this study, we observed a significant reduction in GMV in the right gyrus rectus region after two months of strict lockdown measures. The function of the gyrus rectus (a subregion of the orbitofrontal cortex) is not fully understood [51]. Previous studies utilizing meta-analytical decoding have annotated items such as social, mentalizing, and theory of mind to the gyrus rectus, all of which are associated with high-order cognition [52]. Considering that depression, anxiety, and distress are the majority of responses associated with isolated and confined environments, our findings indicate a potential relationship between alterations in the gyrus rectus and the psychological health issues resulting from measures related to the pandemic. Furthermore, even four months after the lifting of the lockdown, there remained a notable decrease in GMV in the right gyrus rectus region compared to the pre-lockdown period, indicating the impact of lockdown on brain structure may extend beyond the duration of the measure itself. This was further supported by the findings of volumetric decreases in DMN at the third scans, specifically in regions such as the PCC, precuneus, and the inferior parietal region of the frontoparietal network. Previous studies have also found that participants who had undergone extensive preparation for the medical residency selection exam showed significant reductions in the PCC and inferior parietal cortex, which may indicate that stress conditions could affect the morphology of brain regions related to the DMN [53]. Moreover, previous research has revealed brain plasticity using T1 following planned interventions [21,22,23]. Our study adds to the understanding of how enclosed environments, stress, and emotional distress can impact the structural atrophy of the brain. It is worth noting that while more brain regions showed a reduction in GMV four months after the lockdown was lifted, this suggests that the four-month follow-up period may not be sufficient to fully assess the long-term effects of lockdown on brain structure. Further investigation is necessary to explore the reversibility of the lockdown-induced decrease in brain volume and the potential for recovery.

Neurobiological models suggest that pinpointing disrupted communication between brain regions can play a key role in understanding the resulting behavioral deficits [54,55]. Therefore, we assessed the lockdown-related FC alterations and observed that the FC between SMN/ATN and DMN, as well as FC within SMN, were significantly decreased immediately after the lockdown period, and this trend was reversed after four months of deconfinement. Moreover, the changed FC (Scane1–Scane3) between DMN and SMN/ATN, indicating the recovery of FC before and after the lockdown, were found to have a significant correlation with the improved mental status recovery. The DMN, composed of brain regions active during rest and less active during task engagement, includes crucial areas such as the PCC and SFG, responsible for internal control mechanisms, memory retrieval, belief formation, theory of mind, and self-referential processing [56,57]. Conversely, the primary motor cortex and somatosensory cortices primarily contribute to the execution and regulation of voluntary movements, as well as the processing of external sensory information, encompassing tactile sensations, pressure, temperature, pain, and proprioception [58]. One possible explanation for the observed changes in FC is that the prolonged isolation and reduced physical activity may have disrupted the usual interplay between the body and mind, leading to altered self-reflective processes regarding the state of the body. This interpretation is consistent with prior research showing that posterior hubs of the DMN can modulate the reduction of conscious awareness in response to somatosensory stimulation [59,60]. Earlier research has also demonstrated that the FC between the posterior DMN and SMN is significantly related to emotions such as anxiety and depression [61].

The current study also revealed a significant increase in anxiety and depression among college students residing in closed dormitory conditions for about two months compared to after four months of lockdown lifting. These findings could serve as indicators of the negative emotional consequences arising from social isolation, uncertainty during the pandemic, and physical inactivity [62,63]. Existing evidence supports the positive impact of physical activity, exercise, and aerobic fitness on overall well-being and the reduction of mental health disorders [64,65,66]. Engaging in structured physical activities has been demonstrated to alleviate symptoms of anxiety, restlessness, irritability, and aggression in individuals [67]. Additionally, a previous study showed that walking exercise increases FC within the DMN and frontal executive network [68]. A recent neuroimaging study using the Adult-Decision Making Competence battery method suggests that individuals with sedentary behavior may experience a reduction in cognitive control abilities, leading to a greater inclination towards automatic information processing. This study further identified that the influence of sedentary time on decision-making competence was mediated by the global/local efficiency of the dorsal ATN [69]. Therefore, we speculate that the alterations in FC between the posterior DMN and the somatosensory and motor cortices observed in this study may be associated with prolonged sitting during the lockdown period. However, further investigation is needed due to the lack of behavioral measurement related to physical activity and the correlation analysis between functional changes. Taken together, the observed aberrant connectivity in multiple hierarchical systems right after the lockdown period reflects abnormal information transmission between the bottom-up processing of the primary systems and the top-down regulation of the higher-order systems.

There were several limitations in this study. First, as this is the first neuroimaging study investigating changes in brain structure and function among college students subjected to stringent lockdown conditions in the real-life events, it is essential to validate these findings in future research. Second, this study lacks a control group of participants who did not experience the lockdown, limiting the ability to attribute observed changes solely to the lockdown. However, the longitudinal design provides valuable insights into within-subject brain changes and recovery dynamics, offering a unique perspective on the neural impact of short-term confinement. Third, this study is the lack of detailed behavioral measurements related to physical activity, which restricts our ability to directly link observed brain changes to physical inactivity. Future studies should integrate such measures to strengthen causal interpretations of these findings. Fourth, the current study involved a relatively modest sample size; however, it is similar in size to other recent longitudinal studies with two or three data collection points [29,61,70]. Larger sample sizes would have provided more robust insights into the observed structural and functional alterations, as well as more comprehensive clinical evaluations. Finally, it is important to acknowledge that fMRI measures a surrogate signal, which does not directly reflect actual neural activity, similar to other hemodynamic-based imaging techniques [71].

## 5. Conclusions

Utilizing both structural and functional MRI datasets, along with systematic evaluations of participants before, right after, and four months after the lockdown period, our study uncovers that neurological changes may be influenced by social effects in addition to the direct impacts of COVID-19 infection, further supporting the concept of mental contagion within the context of the COVID-19 pandemic. Notably, brain plasticity related to functional connectivity is particularly intriguing, suggesting a significant recovery potential within the human brain connectome. However, the gradual nature of brain structural changes warrants further investigation with longer follow-up periods. The current findings provide valuable insights into the effects of prolonged isolation and reduced physical/social activities on the human brain. Additionally, our results offer potential directions for rehabilitation mechanisms and interventions for individuals exposed to similar physical and social conditions.

## Figures and Tables

**Table 1:** Clinical symptoms in participants right after lockdown and four months after lockdown.

Clinical Symptoms	Scan2 (N = 27)	Scan3 (N = 27)	T Value	*p*-Value ^a^
PSS14	27.5 (9.6)	22.0 (6.9)	3.00	0.0057 **
GAD7	6.3 (4.9)	3.7 (2.5)	2.39	0.0239 *
PHQ9	8.0 (4.8)	4.2 (3.1)	3.85	0.0007 **
PANAS (P)	24.0 (7.0)	26.4 (5.7)	−1.98	0.0583
PANAS (N)	18.6 (8.0)	18.0 (5.4)	0.29	0.7729

^a^ *p*: Paired *t*-test; ** *p* < 0.001, * *p* < 0.05.

**Table 2:** Comparisons of GMV between pairwise groups.

	Region No.	Region	T Value	*p*-Value	Cohen’d	Network
Scan1 vs. Scan2	24	REC_R	6.48	0.00002	0.36	Limbic
	50	CUN_R	4.02	0.0015	0.30	Visual
Scan1 vs. Scan3	17	OLF_L	3.93	0.0024	0.21	Limbic
	24	REC_R	3.59	0.0043	0.39	Limbic
	39	PCC_L	3.76	0.0032	0.34	Default
	65	IPG_L	3.64	0.0039	0.31	Frontoparietal
	67	SMG_L	3.68	0.0036	0.17	V-Att
	71	PUCN_L	3.67	0.0037	0.35	Default

## Data Availability

The data presented in this study are available on request from the corresponding author. The data are not publicly available due to privacy.
